# Therapeutic Efficacy of Different Bladder Monotherapies Versus Multimodal Therapy in Patients with Interstitial Cystitis/Bladder Pain Syndrome

**DOI:** 10.3390/biomedicines14040834

**Published:** 2026-04-06

**Authors:** Wan-Ru Yu, Jia-Fong Jhang, Yuan-Hong Jiang, Hann-Chorng Kuo

**Affiliations:** Department of Urology, Hualien Tzu Chi Hospital, Buddhist Tzu Chi Medical Foundation and Tzu Chi University, Hualien 970, Taiwan; wanzu666@gmail.com (W.-R.Y.); alur1984@hotmail.com (J.-F.J.); redeemerhd@gmail.com (Y.-H.J.)

**Keywords:** cystitis, treatment, bladder inflammation, bladder pain syndrome

## Abstract

**Purpose**: This study compared the therapeutic efficacy of different bladder monotherapies and multimodal therapy in patients with interstitial cystitis/bladder pain syndrome (IC/BPS). **Materials and methods**: In total, 190 patients with a confirmed diagnosis of IC/BPS were treated with different bladder therapies. The bladder monotherapies included intravesical platelet-rich plasma (PRP) injection (*n* = 60), intravesical botulinum toxin A (BoNT-A) injection (*n* = 33), intravesical hyaluronic acid (HA) instillation (*n* = 36), and low-energy shock wave (LESW) bladder therapy (*n* = 61). Multimodal therapy (MMT) was provided to patients who had unsuccessful initial bladder treatment targeting chronic inflammation, urothelial dysfunction, bladder pain, pelvic floor muscle pain, psychological stress, and lower urinary tract dysfunction. The treatment outcome was assessed using self-reported Global Response Assessment scores at 3 months and during the follow-up time points after bladder treatment. **Results**: Thirty-one patients received MMT. The 3-month success rates of bladder therapy were 55.0% for PRP injection, 57.6% for BoNT-A injection, 50.0% for HA instillation, 46.7% for LESW bladder therapy, and 58.1% for MMT. The success rates of bladder monotherapy decreased after 6 months. However, the success rate of MMT increased at 9 (67.7%) and 12 (73.1%) months. Patients treated with MMT exhibited improvement in glomerulation grade after cystoscopic hydrodistention. Only patients with successful treatment outcomes after MMT had improvement in bladder pain severity and pelvic floor muscle pain parameters. **Conclusions**: Bladder monotherapy such as PRP injection, BoNT-A injection, HA instillation, and LESW bladder therapy had successful treatment outcomes in patients with IC/BPS. In patients who had unsuccessful initial bladder therapy, the 3-month success rate of MMT was 58.1% and sustained improvement with time, particularly in the improvement of bladder pain and PFM pain severity.

## 1. Introduction

Interstitial cystitis/bladder pain syndrome (IC/BPS) is a bladder disorder without a definite etiology. Currently, IC/BPS lacks a treatment that is consistently effective and durable for all patients [1]. The clinical symptoms vary widely, and bladder condition and extra-bladder dysfunction also exhibit different clinical presentations in patients with IC/BPS [2]. Previous studies on the pathophysiology of IC/BPS have revealed that IC/BPS is associated with multiple etiological factors affecting bladder condition and clinical symptoms. These include viral or bacterial infection, autoimmune response, mast cell activation, urothelial dysfunction, neurogenic inflammation, and central sensitization [3]. Several non-pharmacological, medical, intravesical, and emerging bladder therapies have been proposed. However, the efficacy and durability of these treatments have not been well elucidated [4].

The principles of IC/BPS treatment involve restoring urothelial function, inhibiting neurological hyperactivity, suppressing inflammation, and managing pain [5]. An essential initial step is determining whether IC/BPS is bladder-centric or non-bladder-centric and whether it represents Hunner’s IC (HIC) or non-Hunner’s IC (NHIC), as the initial management strategies differ significantly between these two subtypes [6]. Therefore, to identify the presence of HIC lesions, patients presenting with intractable bladder pain and reduced bladder capacity should undergo office-based cystoscopic examination without anesthesia [7].

Among various established and emerging bladder therapies for IC/BPS, intravesical hyaluronic acid (HA) instillations have been widely applied to replenish the defective glycosaminoglycan of the bladder urothelium [8]. Intravesical botulinum toxin A (BoNT-A) injections have been recommended to effectively alleviate bladder pain and restore urothelial integrity via its anti-inflammatory effects and facilitate urothelial regeneration [9]. Autologous platelet-rich plasma (PRP) is rich in several growth factors and cytokines that regulate the process of inflammation and regeneration in wound healing process. Recent preliminary studies have shown that intravesical PRP injections improve IC symptoms and yield a satisfactory success rate at 3 months after treatment [10]. In addition, low-energy shock wave (LESW) bladder therapy has been considered as a regenerative medicine for IC/BPS [11]. However, these treatments have not been shown to provide durable treatment outcomes for IC/BPS. Patients with IC/BPS commonly present with anxiety and depression. However, combined cognitive behavioral therapy and bladder therapy can improve IC symptoms [12]. Moreover, pelvic floor muscle pain (PFMP) and voiding dysfunction attributed to urethral sphincter dysfunction or poor relaxation of the pelvic floor muscle are frequently observed in these patients [13].

Bladder monotherapy might not effectively improve IC symptoms. Therefore, the American Urological Association guidelines have recommended multimodal therapy with the combination of several different bladder and extra-bladder therapies [6]. By integrating different treatment modalities, patients who have unsuccessful previous bladder monotherapy may have improved treatment outcomes. Multimodal therapy (MMT) has been suggested based on the possible pathological mechanisms. However, the most appropriate therapeutic strategy has not been well defined [3]. The main reason is that several different pathophysiological mechanisms might be associated with clinical symptoms and bladder conditions [1]. Therefore, the therapeutic strategy should be based on the pathophysiology and clinical phenotypes of IC/BPS, which enables urologists to establish a rational algorithm for concomitant multimodal therapy for IC/BPS [3]. Based on the pathophysiology and clinical characteristics of the bladder disease, it is reasonable to implement a multimodal therapy for patients with IC/BPS and those who present with bladder and extra-bladder pathophysiology [9].

This study compared the therapeutic efficacy of different bladder monotherapies and multimodal therapy in patients with IC/BPS. The study results may provide useful criteria for patients with IC/BPS who can be candidates for multimodal therapy at the start of treatment.

## 2. Methods

This is a proof-of-concept, retrospective analysis of the therapeutic results in a group of patients with IC/BPS who had unsuccessful treatment result to previous bladder monotherapy and received MMT. In total, 190 patients with IC/BPS were treated with different bladder therapies for clinical symptoms at a tertiary center in eastern Taiwan. All patients had a diagnosis of IC/BPS confirmed via cystoscopic hydrodistention under anesthesia. Further, they underwent videourodynamic study to rule out other lower urinary tract dysfunctions. With the confirmation of IC/BPS, the patients were divided into the HIC and NHIC groups. Transurethral electrofulguration or laser ablation of Hunner’s lesions was performed after the diagnosis of HIC. The patients with NHIC were subsequently treated with intravesical HA instillations. If the IC symptoms stabilized or improved, the patients were regularly followed-up at the out-patient clinic. If the IC symptoms did not improve or flared up after the initial treatment, the patients were managed with the second-line bladder treatment.

The bladder monotherapy selected for the patients was not randomized but was introduced after shared decision-making based on a patient’s bladder condition according to previous clinical studies [9]. The bladder monotherapy included intravesical PRP injection (*n* = 60), intravesical BoNT-A injection (*n* = 33), intravesical HA instillations (*n* = 36), and LESW bladder therapy (*n* = 61). The therapeutic results were assessed after the completion of bladder monotherapy. Patients who had a satisfactory response to the bladder monotherapy, or those who had an unsuccessful result but were declined for MMT, continued the same treatment in the follow-up period. The patients who still had persistent or exacerbated IC symptoms after the initial bladder treatment were considered as unsuccessful. MMT was recommended and provided at least 3 months after the initial bladder treatment according to the clinical assessments and concomitant treatment regimens targeting chronic inflammation, urothelial dysfunction, bladder pain, pelvic floor muscle pain, psychological stress, and lower urinary tract dysfunction [3] (Table 1).

### 2.1. Intravesical PRP Injection

The PRP was prepared with 50 mL of whole blood, centrifuged with a soft spin (190× *g*, 20 min, <20 °C), and then further centrifuged by a hard spin (2000× *g*, 20 min, <20 °C). Platelet pellets were added to platelet-poor plasma to form 12 mL of sterile PRP. In total, 10 mL of PRP was used for intravesical injections [14]. The final PRP concentration was approximately 3–4 times the peripheral blood platelet count. The PRP solution was administered via 20 suburothelial injections into the bladder wall during each treatment session, under intravenous general anesthesia, maintaining the bladder volume at 100 mL to facilitate needle injections. Cystoscopic hydrodistention was performed for 15 min after PRP injections. The maximum bladder capacity (MBC) and glomerulation grade were identified after the release of intravesical pressure. The patients underwent four monthly PRP injections, and the follow-up time point was set at 3, 6, 9, and 12 months after the first PRP injection.

### 2.2. Intravesical BoNT-A Injection

The intravesical BoNT-A injection for IC/BPS was performed using a method described in a previous study [15]. In total, 100 units of onabotulinumtoxinA (Allergan, Irvine, CA, USA) was dissolved to form a 10 mL solution with normal saline. The BoNT-A solution was administered via 20 suburothelial injections into the bladder wall during the treatment session, with each injection site receiving 0.5 mL of BoNT-A (5 units), under intravenous general anesthesia, maintaining the bladder volume at 100 mL to facilitate needle injection. Cystoscopic hydrodistention was performed for 15 min after BoNT-A injections, and the MBC and glomerulation grade were determined after the release of intravesical pressure. The follow-up time points were set at 3, 6, 9, and 12 months after the BoNT-A injection.

### 2.3. Intravesical HA Instillation

In patients who had been diagnosed with IC/BPS and who exhibited a characteristic glomerulation formation after hydrodistention, intravesical HA instillations were provided as the first-line bladder therapy. In total, 50 mL of Cystistat@ solution (Myla, Galway, Ireland) containing 40 mg of HA was instilled into the bladder after catheterization and bladder emptying. The treatment was carried out once per week four times and then monthly for 5 more months [16]. The HA solution was retained in the bladder for >2 h unless the patient could not tolerate the bladder fullness. Antibiotics were prescribed to patients who developed micturition pain. The follow-up time points were set at 3, 6, 9, and 12 months after the first HA instillation.

### 2.4. LESW Bladder Therapy

The patients were placed in the supine position, and the bladder was distended with 50–100 mL of urine detected via transabdominal ultrasonography. The shock wave applicator (LITEMED LM-ESWT-mini system, Lite-Med Inc., Taipei, Taiwan) was gently placed directly on the ultrasound transmission gel over the skin surface of the suprapubic region above the urinary bladder once a week for 4 weeks, with 3000 shocks, a frequency of 3 pulses per second, and a maximum energy flow density of 0.25 mJ/mm^2^. The focus zone penetration depth was within 20–150 mm, which indicated that this wide-focused shock wave could be placed easily in the bladder from the suprapubic area. The patient was subjected to LESW delivered to the suprapubic region at five points, as previously reported [17]. The position of the shock wave transducer was changed after every 400 pulses. The procedures were performed without anesthesia or preoperative antibiotics. The follow-up time points were set at 3, 6, 9, and 12 months after the first LESW treatment.

### 2.5. Multimodal Therapy

Patients who did not have a successful treatment outcome after previous monotherapy for IC symptoms received the multimodal therapy at least 3 months after the last bladder treatment. All patients should be assessed again for the bladder condition via cystoscopic hydrodistention (MBC, glomerulations, and the presence of Hunner’s lesion) and other lower urinary tract dysfunctions via videourodynamic study for the presence of bladder hypersensitivity or bladder outlet dysfunction. Further, they should complete all the questionnaires such as the O’Leary Symptom Score, which include interstitial cystitis symptom index (ICSI) and problem index (ICPI). Beck Anxiety Index (BAI), Beck Depression Index, and pelvic floor muscle (PFM) examination were performed to identify PFM tenderness points, determine the most severe tender point, and assess overall PFM pain using a visual analog scale (VAS).

**Figure 1:**
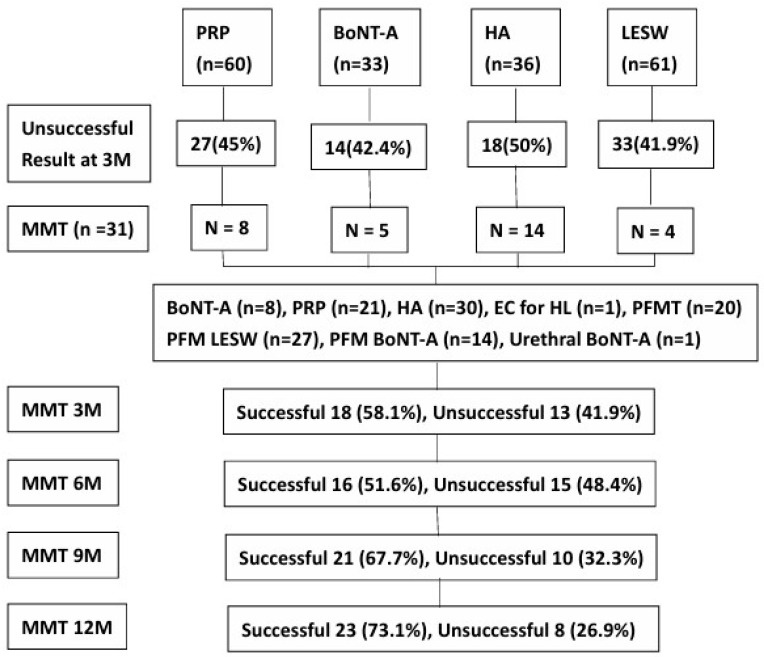
The flow diagram showing the distribution of 190 patients with interstitial cystitis/bladder pain syndrome initially treated with different bladder monotherapy and followed by multimodal therapy (MMT). Because patients were treated with individualized MMT regimen, only the number of patients who received different item of MMT and the percentage of patients with successful result at time points are shown. PRP: platelet-rich plasma intravesical injection, BoNT-A: botulinum toxin A intravesical injection, HA: hyaluronic acid instillations, LESW: low energy shock wave, EC: electrocauterization, HL: Hunner’s lesion, PFMT: pelvic floor muscle physiotherapy.

After the clinical assessments, the patients were treated with concomitant bladder and extra-bladder therapy according to the presence of the positive assessment items. The treatment options for IC/BPS involved a combination of the following approaches: (1) nonsteroidal anti-inflammatory drugs, analgesics, antihistamines, and prednisolone in patients with an IC symptom score ≥12; (2) intravesical BoNT-A injection in patients with a bladder pain VAS score ≥ 5; (3) intravesical PRP injections in patients who developed glomerulation grade ≥ 2 after hydrodistention; (4) bladder instillation of HA in patients with any grade glomerulation; (5) electrofulguration of Hunner’s lesion, with or without PRP or triamcinolone injection, in patients with Hunner’s lesions; (6) antiviral therapy for patients with HIC and suspected viral infection; (7) PFM physiotherapy, PFM BoNT-A injections for patients with PFM focal tenderness; (8) LESW or PFM physiotherapy for patients with PFMP; (9) urethral sphincter BoNT-A injections in patients with urethral sphincter dysfunction; (10) oral antianxiety or antidepressant medications in patients with significant anxiety and depression; (11) medications or antihistamines in patients with hypersensitive bladder; and (12) alpha-blockers and baclofen in patients with voiding dysfunction [3]. The flow diagram of the distribution of 190 patients with IC/BPS initially treated with different bladder monotherapy and the number of patients who received different MMT regimen are shown in Figure 1.

### 2.6. Assessment of Treatment Outcomes

In the subjective assessment, the treatment outcome (primary endpoint) was assessed using the self-reporting Global Response Assessment (GRA) score at the follow-up time points after bladder treatment. Because the treatment outcome of MMT involved not only bladder symptoms, but also extra-bladder condition and psychometric compartment, GRA was chosen as the primary end-point. A successful outcome was defined as a reported GRA score indicating moderate (+2) or significant improvement (+3) [18]. In contrast, the treatment outcomes were considered as unsuccessful if the patients did not achieve this level of improvement [19]. The secondary endpoints included the ICSI and ICPI, which were assessed based on self-reported IC/BPS severity and the numerical rating scale for bladder pain severity. The ICSI ranged from 0 to 20 points, and the total ICPI score was 0–16. Bladder pain was scored using a self-assessed 11-point numerical rating scale for pain severity, and the highest score indicated a serious pain severity.

Psychological stress has been found to contribute to the pelvic pain perception and disease severity of IC/BPS. The psychological stress may exacerbate the pain perception in the bladder and pelvic floor, whereas improvement of pelvic pain may also reduce the psychological stress after bladder and extra-bladder therapy [20]. In this study we also measure the psychometric assessments including personality assessments by type D scale (DS14), the item 16 of interpersonal support evaluation list (ISEL-16), and Chen’s perceived stress scale (PSS). Anxiety severity was also recorded using BAI. A BAI score of 0–18 indicated mild anxiety; 19–29 points, moderate anxiety; and 30–63, severe anxiety [21]. The follow-up time points are set at 3, 6, 9, and 12 months after the initiation of bladder therapy. The patients who had sustained therapeutic efficacy were continued to be followed-up untill 12 months. The patients who did not have a successful treatment outcome at any follow-up time point were recommended to switch to MMT.

### 2.7. Statistical Analysis

Continuous variables were expressed as mean ± standard deviation, while categorical variables were presented as counts and percentages. The comparison of GRA success rates across five treatment subgroups was performed by the chi-square test. Differences in clinical data between the successful and unsuccessful subgroups, as well as changes in variables after bladder monotherapy and MMT, were analyzed using one-way analysis of variance. Post hoc analysis among different treatment subgroups was performed by Bonferroni test. Paired-t test was used to analyze within-group significant differences in variables before and after treatment. A *p*-value < 0.05 was considered statistically significant. Statistical analyses were performed using Statistical Package for the Social Sciences, version 20.0 (IBM Corp., Armonk, NY, USA). Because the study was characterized as a hypothesis-generating study, a formal power calculation of the sample size of MMT group and for detecting meaningful between-group differences was not performed.

This is a retrospective analysis of previously treated patients with IC/BPS. The patients included in this analysis were followed by the same diagnostic criteria, treatment courses, and follow-up protocol. This study had been approved by the Institutional Review Board and Ethics Committee of the Institution (approval No.: 115-015 B, dated 5 February 2026). No informed consent was obtained due to the nature of retrospective analysis. All patients had been informed about the rationale of the treatment and any adverse event was recorded.

## 3. Results

Table 2 shows the baseline demographic characteristics and clinical assessment results of the participants. In total, 31 patients (29 women and 2 men; 30 NHIC and 1 HIC) who had received previous bladder monotherapy underwent MMT for persistent or recurrent IC symptoms. The patients of the MMT group were included in the PRP injection (*n* = 8), BoNT-A injection (*n* = 5), HA instillation (*n* = 14), and LESW bladder therapy (*n* = 4) subgroups. The MMT and other bladder monotherapy subgroups did not significantly differ in terms of age, sex, bladder condition, and IC symptoms.

Table 2 presents the changes in IC symptom scores, functional bladder capacity, and uroflowmetry parameters after different bladder therapies. The success rates of bladder therapy were 55.0% for PRP injection, 57.6% for BoNT-A injection, 50.0% for HA instillation, and 46.7% for LESW bladder therapy. The success rate of MMT was 58.1%, and 31 patients from the other bladder therapy groups had unsuccessful outcomes.

Table 3 depicts the success rates (GRA score of 2 or 3) of bladder monotherapy and multimodal therapy at different timepoints, from 3 to 12 months. Patients who received another type of treatment after the initial bladder therapy were considered to have an unsuccessful outcome. In the bladder monotherapy subgroups, which included PRP injection, BoNT-A injection, HA instillation, and LESW bladder therapy, the success rates decreased after 6 months. However, the success rate of the MMT group increased at 9 (67.7%) and 12 (73.1%) months.

Table 4 shows the changes in IC symptom scores, voiding diary, bladder conditions, and PFM pain parameters from baseline to 3 months between patients who had successful and unsuccessful treatment outcomes after MMT. As shown in Table 4, patients with both successful and unsuccessful treatment outcomes had improvements in symptom scores and glomerulation grade after MMT. However, only patients with a successful treatment outcome exhibited improvements in bladder pain VAS score and PFM pain parameters.

## 4. Discussion

The current study showed that bladder monotherapy such as PRP injection, BoNT-A injection, HA instillation, and LESW bladder therapy had similar successful treatment outcomes in patients with IC/BPS. In patients who had unsuccessful initial bladder therapy, MMT can provide a 3-month success rate of 58.1%, particularly in the improvement of bladder pain and PFM pain severity. The success rates of patients treated with PRP injection, BoNT-A injection, HA instillation, and LESW monotherapy decreased with time. However, the therapeutic efficacy was sustained at 9 and 12 months in the MMT group.

The fundamental pathophysiological mechanisms underlying IC/BPS are bladder inflammation and inflammation-induced increases in apoptosis and urothelial deficiency [22]. Hence, anti-inflammatory therapies and urothelial barrier protein supplementation remain the cornerstone of treatment. However, the long-term efficacy of anti-inflammatory medical treatments has generally been disappointing [5]. Immunohistochemical analysis of the bladder mucosa in patients with IC/BPS has revealed a decrease in the expression of adhesive protein E-cadherin and tight junction proteins within the urothelium [23]. Intravesical instillation of glycosaminoglycan supplements have been used to restore the bladder’s barrier function. However, patients may commonly experience relapse of IC symptoms immediately after the discontinuation of glycosaminoglycan replenishment. This is because urothelial dysfunction has not been completely recovered, most likely because chronic inflammation in the IC/BPS bladder has not been resolved [22].

Chronic inflammation-induced sensory hyper-innervation is a mainstay pathophysiological mechanism underlying the IC/BPS symptoms [22]. Intravesical BoNT-A injections have been used to manage chronic inflammation and inhibit inflammation-induced apoptotic pathways [24]. Repeated intravesical BoNT-A injections every six months also reduce glomerulation severity during cystoscopic hydrodistention [25]. Currently, BoNT-A injection has resulted in improvements in both clinical symptoms and urinary biomarker levels. A previous study has revealed that BoNT-A injection three or four times every 6 months is required to achieve long-term success [15]. Urothelial defects can be adequately repaired only if chronic inflammation has been resolved.

Urothelial deficits and the loss of umbrella cells are closely associated with chronic inflammation in IC/BPS bladders [23]. Notably, impaired urothelial cell proliferation has shown improvement after repeated intravesical PRP injections [26]. Preliminary findings showed significant improvements in bladder pain, irritative symptoms, and bladder capacity after repeated PRP treatments [14]. Further, repeated PRP injections led to significant changes in the expression of proteins related to urothelial cell proliferation, cytoskeletal structure, apoptosis, inflammation, and barrier function in IC/BPS bladders [26].

LESW has emerged as a novel treatment option for patients with IC/BPS who are refractory to conventional medications or glycosaminoglycan supplemental therapies [17]. LESW has anti-inflammatory effects, can enhance local blood flow, and can promote tissue regeneration [11]. Preliminary clinical trials of patients with IC/BPS have reported reductions in bladder pain severity, decreased daytime urinary frequency, and increased voided volumes after LESW therapy [17]. Currently, the frequency and duration of LESW treatment have not been well documented. Based on the low success rate and short therapeutic duration, four-weekly LESW treatments might not be sufficient to explain chronic inflammation and induce urothelial regeneration.

The abovementioned bladder monotherapies have long been applied in patients with IC/BPS. However, their long-term durability remains suboptimal in most patients. These bladder-targeted monotherapies can address specific aspects of the IC/BPS pathophysiology. However, they did not comprehensively resolve the multifactorial nature of the disease, thereby limiting durable treatment outcomes, particularly in refractory cases. In response to the complex and heterogeneous nature of IC/BPS, the latest American Urological Association guidelines have revised the management recommendations, disregarding tiered treatment levels in favor of a multimodal therapeutic approach tailored to identifiable pathophysiological mechanisms [6]. An MMT strategy for IC/BPS should concomitantly integrate various bladder therapies to address the diverse pathophysiological mechanisms. Patients may concurrently receive combined therapies to optimize symptom control and disease management [27].

Patients with IC/BPS, particularly those with non-bladder-centric IC/BPS, frequently present with myofascial pain syndrome [28]. Neuromuscular dysfunction of the PFM is common in both bladder-centric and non-bladder-centric IC/BPS [2]. In patients with severe PFMP and multiple trigger points, a combination of manual PFM massage physical therapy and bladder therapies in MMT can achieve a better treatment outcome. This study showed patients who had successful outcomes exhibited significant improvements in bladder pain and PFMP severity. Continued pelvic floor physiotherapy may provide a long-term therapeutic efficacy in relieving PFM pain and IC bladder symptoms [29].

Psychological stress is strongly associated with symptom severity in patients with IC/BPS, particularly in those who are refractory to conventional therapies. Previous studies have shown that patients with severe IC/BPS often exhibit higher levels of anxiety and depression [30]. Interestingly, the anxiety and depression scales measured by BAI and BDI did not change in the successful MMT subgroups. The psychometric parameters DS14 and ISEL16 also showed no significant change and difference between unsuccessful and successful MMT subgroups. In contrast, PSS showed significant decrease in the successful MMT subgroup and was associated with the improvement of PFM pain severity, suggesting the PFM pain may play a major role in the psychological stress in patients with IC/BPS. Psychiatric interventions primarily alleviate the central perception of bladder discomfort without altering bladder pathology [31]. As shown in the results section of this study, with adequate psychological support in combination with other bladder therapies in MMT, patients with IC/BPS can achieve a better balance between symptom burden and the perception of their bladder status, decreasing the distress caused by this chronic and bothersome disease.

The high long-term successful treatment outcome of MMT in this study has confirmed that the efficacy of combination of multiple bladder and extra-bladder therapies can provide treatment effectiveness in patients who had unsuccessful result to the initial bladder monotherapy. This is because it can cover assessable pathophysiological mechanisms underlying the symptoms of IC/BPS. Extra-vesical conditions such as PFM dysfunction, fasciitis, visceral inflammation, irritable bowel syndrome, endometriosis, and other pelvic organ pathologies can trigger bladder pain and irritative symptoms via a convergent cross talk within the central nervous system [32]. Anxiety, depression, and psychological stress are frequently associated with IC/BPS and can exacerbate symptoms by further disrupting urothelial integrity and triggering symptom flare-ups [33]. MMT targeting bladder mucosal dysfunction, PFMP, and psychological stress could improve the patient’s perception of bladder discomfort along with multiple bladder therapies including BoNT-A injection for pain, PRP injection for urothelial regeneration, and HA instillations for the establishment of urothelial barrier.

There are several limitations of this study. First, this is not a randomized study. The patients who received MMT were those who had unsuccessful treatment result to previous bladder monotherapy, which may introduce substantial selection bias. The comparison of therapeutic results between MMT and monotherapy groups is not a head-to-head comparison, but to provide a proof-of-concept that patients who failed previous bladder monotherapy such as PRP, LESW, BoNT-A, or HA could be improved by MMT and the treatment success could be sustained for a longer period compared to monotherapy, based on their underlying pathophysiology and clinical characteristics. Second, the case number of MMT group was small, which might also result in statistical bias. However, because there are multiple treatment modalities in the same treatment course, patients might not accept the complicated management for their bladder disease. Further randomized and longitudinal study is needed to verify the therapeutic efficacy of this treatment.

## 5. Conclusions

Combining multiple bladder and extra-bladder therapies that target chronic inflammation, bladder pain, urothelial dysfunction, psychological stress, and PFM pain can provide a satisfactory success rate in patients with IC/BPS who previously had unsuccessful bladder monotherapy, including intravesical PRP injection, BoNT-A injection, or LESW bladder therapy and HA instillation. The success rate of MMT can also increase and be sustained for longer period. To address all underlying pathophysiological mechanisms, which can help achieve a better therapeutic success, patients with IC/BPS should be comprehensively investigated upon diagnosis and treated with multiple modalities.

## Figures and Tables

**Table 1:** Recommended treatment modalities selected based on the clinical assessment results.

Clinical Assessment	Criteria	Treatment Modality
1. IC symptom score	ICSI ≥ 12	NSAID, analgesic, narcotic drugs × 3 M
2. Bladder pain VAS score (0–10)	≥5	Intravesical BoNT-A 100U injection × 1
3. Glomerulation after HD	≥Grade 2	Intravesical PRP injections Q1M × 4
4. Glomerulation after HD	Any grade	Bladder instillation of HA × 9 times
5. Presence of Hunner’s lesion	Yes	Electrofulguration or laser ablation of Hunner’s lesions
6. Hunner’s lesion with virus infection	Elevated EBV title	Antiviral agent, Valacyclovir
7. Pelvic floor muscle focal tenderness	VAS ≥ 3	PFM BoNT-A 100U local injection × 1 PFM physiotherapy
8. Pelvic floor muscle tenderness	Any grade	LESW of PFM and PFM physiotherapy
9. Urethral sphincter dysfunction	Yes	Urethral sphincter BoNT-A 100U injection × 1
10. Anxiety and depression	BAI ≥ 18	Antianxiety, antidepressant, or psychiatric consultation
11. Bladder hypersensitivity	Yes	Medication for bladder hypersensitivity
12. Voiding dysfunction	Yes	Alpha-blocker, baclofen

IC: interstitial cystitis, ICSI: interstitial cystitis symptom index; NSAID: non-steroid anti-inflammatory drugs; HD: cystoscopic hydrodistention; BoNT-A: botulinum toxin A; EBV: Epstein–Barr virus; HA: hyaluronic acid; LESW: low energy shock wave; PRP: platelet-rich plasma, PFM: pelvic floor muscle; VAS: visual analog scale for pain; BAI: Beck’s anxiety index.

**Table 2:** Three-month treatment outcomes and changes in the measured variables between different bladder monotherapies and multimodal therapy.

		1. PRP (*n* = 60)	2. BoNT-A (*n* = 33)	3. HA (*n* = 36)	4. LESW (*n* = 61)	5. MMT (*n* = 31)	*p*-Value	Post Hoc
Age		53.6 ± 14.8	54.8 ± 10.1	54.8 ± 12.1	54.1 ± 12.7	53.3 ± 13.1	0.981	
F/M		52/8	28/5	34/2	53/7	28/3	0.736	
MBC (mL)		848 ± 194	856 ± 153	781 ± 220	784 ± 180	782 ± 177	0.161	
Glom		1.34 ± 0.78	1.56 ± 0.67	1.69 ± 0.71	1.55 ± 0.62	1.87 ± 0.76	0.017	1v3, 5; 4v5
GRA ≥ 2		33 (55.0%)	19 (57.6%)	18 (50.0%)	28 (46.7%)	18 (58.1%)	0.760	
GRA < 2		27 (45.0%)	14 (42.4%)	18 (50.0%)	32 (53.3%)	13 (41.9%)		
ICSI	BL	13.0 ± 4.30	14.6 ± 3.89	11.36 ± 4.30	11.3 ± 3.85	13.7 ± 3.68	0.001	1v4; 2v3, 4; 5v3, 4
	3 M	10.3 ± 5.35 *	11.0 ± 5.45 *	10.27 ± 5.00	10.4 ± 4.22	8.90 ± 3.35 *	0.501	
ICPI	BL	12.3 ± 3.19	12.7 ± 3.53	10.72 ± 4.26	10.8 ± 3.76	12.3 ± 3.08	0.029	1v3, 4; 2v3, 4
	3 M	9.23 ± 4.33 *	9.27 ± 4.33 *	9.46 ± 5.23 *	9.15 ± 3.92 *	8.52 ± 3.60 *	0.927	
OSS	BL	25.3 ± 7.01	27.2 ± 7.17	22.1 ± 8.25	22.1 ± 7.21	26.0 ± 6.50	0.002	1v3, 4; 2v3, 4; 5v3, 4
	3 M	19.5 ± 9.35 *	20.3 ± 9.51 *	19.7 ± 9.98 *	19.5 ± 7.88 *	17.4 ± 6.56 *	0.743	
VAS	BL	4.48 ± 3.00	6.12 ± 3.51	3.94 ± 2.94	4.15 ± 3.04	5.94 ± 2.39	0.002	1v2, 5; 2v3, 4; 5v3, 4
	3 M	3.02 ± 2.83 *	4.03 ± 3.39 *	3.23 ± 3.04	3.12 ± 2.66 *	3.61 ± 2.29 *	0.512	
FBC (mL)	BL	259 ± 101	299 ± 113	-	313 ± 144	291 ± 108	0.191	
	3 M	273 ± 122	288 ± 124	-	281 ± 117	268 ± 101	0.919	
Qmax (mL/s)	BL	15.1 ± 7.56	15.9 ± 9.57	14.7 ± 7.65	14.2 ± 7.59	11.1 ± 6.17	0.126	
	3 M	14.2 ± 7.65	14.8 ± 10.4	12.7 ± 7.76	15.2 ± 7.85	13.6 ± 8.25	0.755	
Volume (mL)	BL	201 ± 116	213 ± 118	228 ± 126	219 ± 104	203 ± 116	0.819	
	3 M	224 ± 114	206 ± 142	187 ± 100	216 ± 117	203 ± 100	0.743	
PVR (mL)	BL	22.5 ± 59.5	12.1 ± 17.7	21.0 ± 32.5	14.9 ± 21.5	19.4 ± 25.0	0.671	
	3 M	15.0 ± 22.5	47.2 ± 88.5	11.9 ± 17.5	11.5 ± 17.2	35.3 ± 45.7	0.010	

* Paired-t test was used to analyze within-group significant differences in variables before and after treatment. Post hoc analysis among different treatment subgroups was performed by Bonferroni test. A *p*-value < 0.05 was considered statistically significant. Abbreviations: PRP: platelet-rich plasma, BoNT-A: botulinum toxin A, HA: hyaluronic acid, LESW: low energy shock wave, MMT: multimodal therapy, MBC: maximal bladder capacity, Glom: grade of glomerulation, GRA: global response assessment, BL: baseline; 3 M: 3 months after completed treatment; ICSI: interstitial cystitis symptom index, ICPI: interstitial cystitis problem index, OSS: O’Leary–Sant symptom score, VAS: visual analog scale, FBC: functional bladder capacity, Qmax: maximum flow rate, PVR: post-void residual.

**Table 3:** Rates of successful treatment outcomes at various time points in patients who received different bladder monotherapies and multimodal therapy.

	PRP (*n* = 60)	BoNT-A (*n* = 33)	HA (*n* = 36)	LESW (*n* = 61)	MMT (*n* = 31)
3 months	33 (55.0%)	19 (57.6%)	18 (50.0%)	28 (46.7%)	18 (58.1%)
6 months	22 (36.7%)	22 (66.7%)	16 (44.4%)	21 (34.4%)	16 (51.6%)
9 months	12 (20%)	15 (45.5%)	12 (33.3%)	13 (21.3%)	21 (67.7%)
12 months	9 (15%)	13 (39.4%)	7 (19.4%)	9 (14.8%)	23 (73.1%)

Abbreviations: PRP: platelet-rich plasma, BoNT-A: botulinum toxin A, HA: hyaluronic acid, LESW: low energy shock wave, MMT: multimodal therapy.

**Table 4:** Symptom scores, cystoscopic findings, and voiding diary records at baseline and 3 months after multimodal therapy in patients with successful and unsuccessful treatment outcomes.

	Time	Total Patients	Unsuccessful (*n* = 13)	Successful (*n* = 18)	*p*-Value
ICSI	Baseline	13.7 ± 3.68	13.9 ± 3.86	13.4 ± 3.65	0.728
	3 M	8.9 ± 3.35 *	10.2 ± 3.52 *	7.94 ± 2.96 *	0.059
ICPI	Baseline	12.3 ± 3.08	12.7 ± 2.63	12.1 ± 3.42	0.579
	3 M	8.52 ± 3.6 *	10.6 ± 2.93 *	7 ± 3.31 *	0.004
VAS	Baseline	5.94 ± 2.39	5.77 ± 2.39	6.06 ± 2.46	0.748
	3 M	3.61 ± 2.29 *	4.85 ± 2.51	2.72 ± 1.67 *	0.008
PSS	Baseline	17.9 ± 7.39	19.9 ± 8.09	16.5 ± 6.72	0.219
	3 M	15.3 ± 6.69 *	18.1 ± 6.49	13.3 ± 6.27 *	0.050
DS14	Baseline	23.2 ± 11.1	23.5 ± 11.2	22.9 ± 11.3	0.886
	3 M	23.8 ± 9.88	27.4 ± 10.0	21.2 ± 9.21	0.087
ISEL-16	Baseline	24.3 ± 5.81	25.2 ± 6.07	23.7 ± 5.71	0.491
	3 M	25.3 ± 4.21	23.6 ± 4.13	26.4 ± 3.96	0.064
BAI	Baseline	13.8 ± 11.3	14.4 ± 14.2	13.4 ± 9.16	0.824
	3 M	14.4 ± 11.6	17.9 ± 14.1	11.9 ± 8.99	0.200
BDI	Baseline	15.0 ± 12.7	18.4 ± 15.9	12.6 ± 9.65	0.214
	3 M	13.0 ± 10.6	16.5 ± 11.4	10.4 ± 9.48	0.112
MBC	Baseline	782 ± 177	715 ± 191	831 ± 154	0.073
	3 M	887 ± 160 *	854 ± 195 *	908 ± 133	0.372
Glomerulation	Baseline	1.87 ± 0.76	2.15 ± 0.69	1.67 ± 0.77	0.079
	3 M	1.06 ± 0.85 *	1.46 ± 0.78 *	0.78 ± 0.81 *	0.025
Frequency	Baseline	12.5 ± 4.93	13.7 ± 6.48	11.6 ± 3.36	0.295
	3 M	11.3 ± 4.34 *	13.5 ± 5.13	9.73 ± 2.9 *	0.015
Nocturia	Baseline	2.24 ± 1.43	2.56 ± 1.85	2 ± 1.04	0.290
	3 M	2.19 ± 1.34	2.75 ± 1.69	1.79 ± 0.85	0.046
FBC (mL)	Baseline	291 ± 108	292 ± 133	291 ± 89.2	0.981
	3 M	267 ± 103	235 ± 93.6 *	291± 105	0.127
PFM tendered score	Baseline	5.0 ± 3.22	4.73 ± 3.74	5.18 ± 2.94	0.726
	3 M	3.46 ± 3.13 *	4.73 ± 3.47	2.65 ± 2.69 *	0.086
PFMP VAS	Baseline	2.18 ± 0.94	2 ± 1	2.29 ± 0.92	0.432
	3 M	1.71 ± 0.85 *	2.09 ± 0.94	1.47 ± 0.72 *	0.059
PFMP points	Baseline	2.61 ± 2.25	2.45 ± 2.5	2.71 ± 2.14	0.779
	3 M	1.46 ± 1.53 *	1.82 ± 1.66	1.24 ± 1.44 *	0.333

* Paired-t test was used to analyze within-group significant differences in variables before and after treatment. A *p*-value < 0.05 was considered statistically significant. Abbreviations: ICSI: interstitial cystitis symptom index, ICPI: interstitial cystitis problem index, VAS: visual analog scale, PSS: perceived stress scale, DS14: type-D personality scale, ISEL-16: Interpersonal support evaluation list, BAI: Beck’s anxiety index, BDI: Beck’s depression index, MBC: maximal bladder capacity, FBC: functional bladder capacity, PFM: pelvic floor muscle, PFMP: pelvic floor muscle pain.

## Data Availability

Data are available on request to the corresponding author.
